# Fish Consumption during Pregnancy in Relation to National Guidance in England in a Mixed-Methods Study: The PEAR Study

**DOI:** 10.3390/nu15143217

**Published:** 2023-07-20

**Authors:** Lucy Beasant, Jenny Ingram, Caroline M. Taylor

**Affiliations:** Centre for Academic Child Health, Bristol Medical School, University of Bristol, Bristol BS8 2PS, UK; lucy.beasant@bristol.ac.uk (L.B.); jenny.ingram@bristol.ac.uk (J.I.)

**Keywords:** diet, pregnancy, guidance, nutrition, fish, mercury, PEAR study, mixed methods

## Abstract

Guidance on foods to limit or avoid in pregnancy is provided on the NHS website for England. Advice on fish consumption is related to exposure to mercury, dioxins and polychlorinated biphenyls, which may have adverse effects on fetal neurodevelopment. Our aim was to provide evidence on the effectiveness of the guidance in minimising exposure to toxins while maximising nutrient intake in a mixed-methods study. An online questionnaire on fish consumption before/during pregnancy was completed by postpartum women (≤12 months) in England (n = 598). A subsample of participants was invited to take part in an interview (n = 14). Women who ate fish before pregnancy reduced their intakes of both oily and white fish during pregnancy, with some avoiding it altogether. Women did not exceed the limit on tinned tuna, but there was evidence of mis-recall on the suggested limit. Overall intakes of fish were below that recommended during pregnancy (36% compliance for pre-pregnancy consumers). Barriers to fish consumption included risk aversion, confusion over specific details of the guidance, cost, availability, family preferences and smell/taste. Clarity and simplicity of the NHS guidance, with an overall message on the number of portions of fish a week advised prominently shown, would help pregnant women to benefit from the nutrients in fish while minimising exposure to toxins. The guidance on the number of cans of tuna advised per week is poorly recalled and needs to be disseminated accurately. The guidance on shark/marlin/swordfish could receive less prominence as it is rarely eaten by pregnant women in England.

## 1. Introduction

During pregnancy, the guidance in England given to women is to follow a healthy diet broadly similar to that advised for the general population [1]. There is additional guidance regarding a number of foods and drinks that pregnant women are advised to either limit consumption of or avoid altogether [2,3,4,5,6]. This guidance is based on several factors including reduction of teratogenic, pharmacological, microbiological and toxicological hazards.

Specifically, through advice on the consumption of types of fish to avoid and/or limit, the guidance aims to limit exposure to the toxic metal mercury, as well as to dioxins and polychlorinated biphenyls. Mercury passes freely though the placenta and has direct neurotoxic effects on the fetus, and/or causes epigenetic changes that may affect the future development of the child. It is associated with a risk of adverse fetal and child developmental effects including neurodevelopmental disorders [7,8,9]. The guidance in England is based on the findings of the UK Scientific Advisory Committee on Nutrition (SACN) on fish consumption in 2004, which uses a calculation of the mercury content of fish that would result in not exceeding a provisional tolerable weekly intake by pregnant women, and be sufficient to protect against the adverse effects on neurodevelopment in the fetus [10]. It includes advice on avoiding predatory fish that contain the highest concentrations of mercury (shark, marlin and swordfish) and on limiting consumption of oily fish (at least one but no more than two portions per week) and tuna (no more than four medium-sized cans per week or two tuna steaks) (Supplementary Table S1).

As well as containing mercury and other pollutants, fish is a rich source of nutrients that are essential for optimal fetal development, including vitamin D, choline, selenium, iodine and long-chain fatty acids, which may at least partly mitigate against the adverse effects of mercury from fish. The beneficial effects of fish consumption on many aspects of maternal and child health, often despite increased levels of maternal blood or hair mercury, are now well established [7,8,9].

The guidance on fish consumption in the UK is complex, requiring an ability to identify oily versus white fish and species of fish, as well as a weekly tally of consumption [2,11]. Similar complex guidance in the USA has been shown to cause pregnant women to avoid fish altogether [12], and in Australian women risk aversion was identified as a major factor in influencing the consumption of fish during pregnancy [13]. Thus risk-averse behaviour in pregnant women may result in them missing out on the beneficial effects of fish on fetal development.

Little is known, however, about the effects of the existing guidance on fish consumption on the frequency of the consumption of fish by pregnant women in England. Indeed, there is little current information on the frequency of consumption of fish in pregnancy in England, but there is evidence that consumption is below the lower limit of two portions of fish a week in total (including at least one portion of oily fish) recommended for the general population and for pregnancy by SACN [10,14,15,16]. Such information could provide an evidence base to inform the future development of the content of the guidance and its dissemination in order to maximise its usability and beneficial impact. The primary aim of this study therefore was to determine compliance with the NHS guidance on the consumption of fish during pregnancy. The secondary aim was to determine the sources of information used by pregnant women to inform themselves about guidance on fish, and which sources they trusted most. To address these aims, this study comprised a quantitative questionnaire with qualitative interviewing in a mixed-methods design with pregnant women living in England.

## 2. Methods

### 2.1. Overall Design

In an explanatory sequential mixed-methods design, recently postpartum women (≤12 months) resident in England for ≥6 months of their pregnancy were first recruited to complete a custom-designed online questionnaire hosted on Jisc Online Surveys [17]. Women who completed the online questionnaire were invited to specify whether they would be willing to be contacted about taking part in an interview. The study is part of a larger study on dietary exposure to toxic metals (The Pregnancy, the Environment And nutRition (PEAR) Study) [18]. As well as the mixed-methods study reported here, the study also includes the recruitment of pregnant women with an in-depth measurement of concurrent dietary intake and collection of samples for analyses of toxic metals.

### 2.2. Questionnaire: Quantitative Data

#### 2.2.1. Development

The initial version of the questionnaire was tested with postpartum women (n = 9) in an adapted ‘Think Aloud’ exercise and modified according to their feedback [19,20]. Participants were emailed a link to access the electronic questionnaire and answered each question in the virtual presence of a researcher (LB). Participants were asked to ‘Think Aloud’ as they accessed and filled in the questionnaire, vocalising their thoughts about the questions, covering, for example, any comprehension issues, the acceptability of available answers, and technical problems including skip rules and the order of questions. Development of the questionnaire was iterative, with alterations being made in response to the comments of up to five participants at a time, until data saturation was reached and no new issues were reported.

#### 2.2.2. Application

The finalised questionnaire was open from April to September 2021. Participants were recruited primarily through publicity with paid advertising boosts on a study Facebook page linked to the study website, with direct access to the questionnaire from the website [18]. With the exception of the screening questions to determine eligibility (resident in England for ≥6 months of pregnancy, ≤12 months postpartum), no questions were compulsory to maximise the completion rate. Participants were able to re-access their partially completed questionnaire so that they did not have to complete it in one session. Questions included those in the following categories.

(1) *Screening questions* (consent, location during pregnancy, age of baby).
(2) *Demographics* (e.g., geographical location, ethnicity, age, highest educational qualification, household income, parity). Where comparable data were available, the values were compared with the most recent values for the population in England to gauge the representativeness of the participants [21,22,23].
(3) *Consumption of fish (before and during pregnancy).* There were three questions in relation to fish consumption (Table 1). The items included were seafood items listed in the NHS website with guidance to avoid or limit during pregnancy because of potential mercury exposure. The questionnaire did not include items that involved guidance on preparation or cooking methods (uncooked shellfish, sushi without freezing the raw fish) or supplements derived from fish oil (for example, cod liver oil). Assessment of compliance with guidance on thoroughly cooking smoked fish or only eating sushi comprising cooked fish was not included as these were added as updates to the guidance after the survey had closed.
(4) *Sources of information about the guidance* (e.g., midwife or other healthcare professional, NHS website, other websites, leaflets, apps, friends and relatives). Participants were also asked to provide free text on which sources of information they trusted and which they felt less confident in. The questions in this section allowed for multiple answers to be given.

### 2.3. In-Depth Interviews: Qualitative Data

Participants were sampled purposively from those that had completed the finalised questionnaire to ensure a range of women in terms of age, ethnicity, educational attainment, and whether or not women limited or avoided fish during their pregnancy. Participants who stated on their completed questionnaire that they were willing to discuss their views and behaviour in more detail were emailed a copy of the participant information sheet at least 24 h before the discussion. Any questions the participants had about their involvement were answered prior to the discussion (either via email or telephone), a convenient time to talk was arranged, and a blank copy of the study consent form was emailed prior to the discussion. Participants were advised not to print/return a copy of the consent form prior to participation because audio-recorded consent would be taken on the day. All interviews were conducted via Zoom video call or telephone by an experienced qualitative researcher (LB) using a semi-structured topic guide (Supplementary Text 1). It was anticipated that each interview would last for 60 min. At the start of each discussion the researcher read each point on the consent form and gained audio-recorded verbal consent from the participant. A separate research discussion was then audio-recorded. No visual recordings were made when discussions were conducted via Zoom. All consent and data audio-recordings were made using an encrypted device, and were then uploaded and stored securely on a University of Bristol server. Towards the end of the discussion a website link was emailed to participants so that they could access current guidance and give their feedback (NHS website for fish consumption in pregnancy) [3]. Participants were emailed a £15 gift voucher to thank them for participating in the interview and a PDF copy of their completed consent form.

### 2.4. Analysis

#### 2.4.1. Questionnaire: Quantitative Data

Data were analysed with IBM SPSS Statistics version 26. The demographic characteristics of the participants who completed the questionnaire were summarised and compared with national data where available. The demographic characteristics of those who completed interviews were also summarised and compared with those of participants who completed the questionnaire. Descriptive statistics were compiled to document and compare the frequencies, before and during pregnancy, of total fish and subtypes of fish and seafood (shark/marlin/swordfish, oily fish, tinned tuna, fresh tuna, white fish, shellfish). Summary descriptive statistics on the reasons for avoiding fish in pregnancy and the main sources of information in pregnancy were compiled.

#### 2.4.2. In-Depth Interviews: Qualitative Data

Anonymised transcripts were coded and organised using NVivo software [24]. Free text comments fed back via the online questionnaire (collecting quantifiable data) were also uploaded to NVivo and integrated into the ongoing qualitative analysis. Analysis of interview transcripts drew upon thematic analysis techniques [25]. LB coded all interview and free text data, and CMT independently coded 25% of the interview data. All data were tabulated and discussed in regular data analysis meetings (LB, CMT) to discuss developing themes and make any adjustments to the topic guide where necessary. Ongoing data collection and analysis was iterative until data saturation was reached, and no new information was found in the data.

## 3. Results

### 3.1. Questionnaire: Quantitative Data

The demographic characteristics of those women who completed the questionnaire were broadly comparable with national indicators for age and home location (Table 2), but they were in general more highly educated and had a greater household income.

When asked about whether there was any difference in fish consumption during pregnancy from before, 4% of pre-pregnancy consumers reported that that although they ate it before pregnancy they avoided it completely during pregnancy, and 27% reported that they ate it less often. When asked about the specific frequency of total fish intake, there was little change in the proportion of women who ate any fish during pregnancy compared with before (83% vs. 84%, respectively), but the overall frequency of consumption declined during pregnancy (Table 3): the percentage eating fish twice a week/more than twice per week before versus during pregnancy in compliance with the SACN recommendation for pregnancy [10] declined from 31% to 26%. The proportion stating that they ate white fish declined very little (3 percentage points), but there was a decline in the consumption of oily fish by 7 percentage points. As with total fish, there was evidence of a decline in the frequency of consumption of both white and oily fish during pregnancy compared with before pregnancy. Very few participants ate shark/marlin/swordfish before pregnancy (8%) and then only infrequently (less than once per month 100%), and the proportion never eating them increased sharply in pregnancy by 7 percentage points (Table 4). There was also a decline in the proportion of consumers of tinned tuna (−7 percentage points) and fresh tuna (−19 percentage points). Consumption of shellfish declined by 25 percentage points.

For those respondents who ate less or avoided fish completely during pregnancy, the main reason was ‘for the health of the baby’ (91%) followed by ‘having no appetite for it’ (48%) or ‘making me feel sick’ (47%). The major source of information on foods to avoid or limit including fish was online (72%), predominantly the NHS website (76% of those who accessed information online), with a high proportion also mentioning midwives as a source of information (68% of those who accessed information by someone telling them and 95% of those who accessed information through a leaflet).

### 3.2. In-Depth Interviews: Qualitative Data

**Figure 1:**
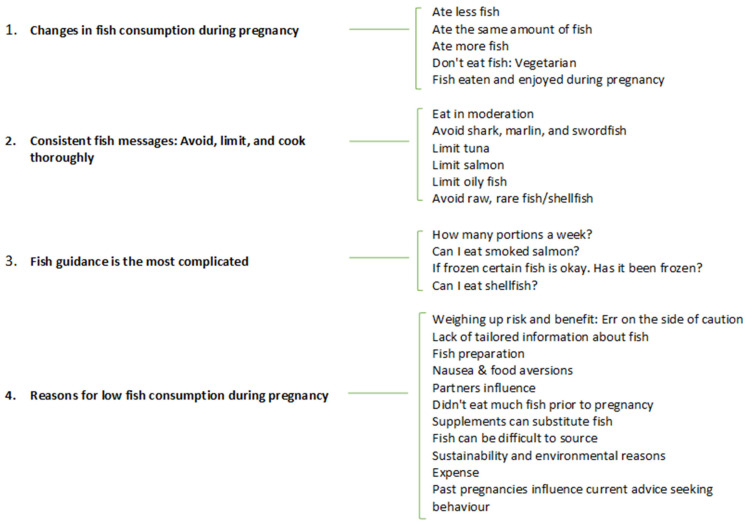
Themes and sub-themes from qualitative in-depth discussions with recently postpartum women in England (n = 14).

Fourteen women took part in an interview between May and December 2021. Twelve women chose to complete the discussion via video call and two by telephone. The participants’ characteristics are shown in Table 1. Those who participated in an interview were more likely to be from the east/Greater London/south east/south west, be multiparous and not do paid work than those who completed the questionnaire, but the differences were only weakly significant (data not shown). Interviews lasted a mean of 47 min (range 33 to 56 min). Women’s experiences are reported in four themes. Themes and sub-themes derived from the analysis are shown in Figure 1. Themes and sub-themes with illustrative quotations can be found in Supplementary Table S2.

#### 3.2.1. Changes in Fish Consumption during Pregnancy

Nearly half of the participants ate less fish during their most recent pregnancy (6/14), four ate the same amount, three ate more fish, and one participant was vegetarian/no fish. Most of those who ate less fish stated that they avoided it for the health of their baby. Those who ate less fish reported that it was “*easier*” to avoid fish entirely (#006), they didn’t want to “*take that risk*” (#005), or were worried that they might “*get it wrong*” (#003). Some who ate less fish felt it was an effort to remember the number of portions consumed in a week “*how many I have had this week*” (#008), and they struggled to *“keep track”* (#006) of the number of portions consumed. Some women reported eating significantly less than the NHS guidance recommends e.g., *“I made sure I limited that [tuna], I only had one can a week”* (#005); *“made sure to only eat salmon once a week”* (#015). Women who ate the same amount of fish were *“more conscious of how and what [fish] I am eating”* (#011), *“because of the levels of whatever is in it”* (#009), or generally felt *“the main things to avoid were probably things I wouldn’t really have eaten much of anyway”* (#010). Only one participant consumed more oily fish than the amount recommended by the NHS guidance during their pregnancy, but this was not an increase in fish consumption since this participant routinely included a high number of fish portions in their diet prior to pregnancy, *“I was ignoring the fact that I shouldn’t be eating more than two portions of oily fish a week”* (#001). Women who reported that they ate more fish during pregnancy, did so for very specific reasons; *“I think mine went up once I had been diagnosed with gestational diabetes”* (#004), *“it was one of the few things that didn’t make me feel sick”* (#002), and the third actively increased their fish intake, *“I felt like… it’s quite a healthy thing to eat”* (#012). The two most popular fish consumed by women were those which they were aware should be limited during pregnancy: salmon and tuna. Cooked shellfish such as prawns were also popular, and some women reported consuming white fish, fish and chips, mackerel, sardines and sushi/poke during their pregnancy.

#### 3.2.2. Salient Fish Messages: Avoid, Limit and Cook Thoroughly

Messages from NHS guidance for fish consumption during pregnancy that were consistently discussed by women, and appeared to be salient in terms of ‘current’ knowledge included messaging that certain types of fish should be avoided, limited or cooked thoroughly during pregnancy. There was a general view that fish should be eaten in “*moderation*” (#009, #010, #014) during pregnancy. Most women recalled that shark, marlin and swordfish should be avoided. Three women recalled information about “*what to avoid*” (#005) as being very prominent, “*on the outside of the plastic folder”* given to them by their midwife (#002). However, all but one stated they never ate these types of fish, *“we were actually [on holiday] and we had marlin”* (#014). Shark, marlin and swordfish were generally perceived as *“unusual” (#006)* fish that are not routinely available or consumed in the UK. Women were also aware that oily fish (specifically salmon) and tuna should be ‘limited’ during pregnancy. Most discussed limiting tuna, and a few recognised that it should be limited because “*you’re not supposed to eat a lot of mercury”* (#015). Others recognised that “*certain types of fish”* (#007) should be avoided or limited because of mercury levels. Most women were also aware that salmon and/or oily fish more generally should be limited during pregnancy, although none discussed reasons for this. Women were also aware that fish and shellfish must be thoroughly cooked, *“we didn’t have raw fish”* (#008). However, some opted to avoid all shellfish due to concerns about increased risk of food poisoning (see ‘Reasons for low fish consumption during pregnancy—Weighing up risk and benefit: Err on the side of caution’). Most women who previously consumed fish had restricted their intake more than the NHS guidance recommends, and for more types of fish than necessary. Few women who ate fish highlighted health benefits of consuming oily fish during pregnancy, and some who discussed health benefits were not sure where they got this information.

#### 3.2.3. Fish Guidance Is the Most Complicated

Although women recalled that oily and tuna fish should be limited, there was confusion when recalling the number of portions that could safely be consumed per week, and NHS guidance on fish consumption was considered the *“most complicated”* (#012). All the women significantly underestimated the amount of tuna they could eat, and restricted their intake to one or two cans per week “*it’s advised not to have more than a couple of tins a week*” (#002). In one case a participant had been advised by a midwife, “*I wouldn’t eat more than two tins a week”* (#003). Very few recalled the limit currently recommended on the NHS website, up to four cans per week, and three were surprised that they could have consumed more when reviewing the guidance at the end of the interview, “*Oh wow, [four cans of tuna] a week, yeah, see I would have thought it was just one”* (#014). Although women were generally more consistent when recalling the number of portions of oily fish that could safely be consumed (no more than two a week), some still reduced their consumption to one portion per week. At times guidance specific to oily fish (no more than 2 portions per week) appeared to be applied as a blanket rule to all types of fish “*you just shouldn’t eat fish more than twice a week*” (#015). Some women were unsure about portion size, “*I suppose two portions is quite subjective*” (#001) or questioned whether the portion information on a packet of fish was for nutritional purposes rather than safety during pregnancy “*you have got portions nutritionally and then portions in terms of what they are allowing you*” (#008). Most women who discussed smoked salmon were confused about whether or not they could eat this during pregnancy, either because they were unsure whether it was considered to be raw fish, or they were following guidance from a previous pregnancy. One participant was particularly confused by NHS guidance because it stated under one heading “*smoked salmon you can eat*”, but “*salmon I think is one of those that you should only have a couple of times a week*” (#004) under the separate heading ‘What to limit’. Women consistently restricted their fish intake more than was necessary, and some avoided or limited fish that they could potentially eat safely without restriction (e.g., cooked shellfish such as prawns) or by following guidance, (e.g., raw or lightly cooked fish in sushi, if the fish has been frozen first). Some women were aware that certain fish was safe to eat if it had been frozen first. However, they still avoided certain fish in these circumstances, particularly if they were not preparing it themselves and could not be 100% sure whether sushi had been frozen (#001, #008). Reasons for low fish consumption during pregnancy are discussed below.

#### 3.2.4. Reasons for Low Fish Consumption during Pregnancy

Behaviours such as limiting or avoiding fish were largely described in terms of weighing up the potential risks and benefits of fish consumption during pregnancy. After reading guidance on fish to limit or avoid, (see Salient fish messages) those who reported eating less fish either felt that it was safer to avoid oily fish, or stop eating certain oily fish that concerned them, e.g., smoked mackerel *“rather be safe than sorry”* (#008) or shellfish e.g., *“prawns or mussels”* (#005). One participant who lived by the sea and had eaten shellfish weekly prior to pregnancy (*“one of my friends owns cockle sheds”* #005) cut shellfish out completely, *“I don’t want to even take that risk … if I can eat something else then I will just eat something else”* (#005). Concern about food poisoning from fish was mentioned by most of the participants, with some wanting clearer guidance on *“what is food poisoning going to do?”* (#015) and the associated risk *“Is it a situation where every time I eat an oyster there’s a 50/50 chance of a miscarriage?”* (#015). Even women who felt they ate more or the same amount of fish during their pregnancy erred on the side of caution “*I don’t trust that it [shellfish] will be cooked properly*” (#012). Others substituted smoked for cooked salmon (#011), made sure fish was *“cooked properly”* (#010) or purchased fish from *“reliable sources”* (#011). Most woman researched foods to limit or avoid themselves online early in their pregnancy, prior to their booking appointment, but women reported very few positive messages about the nutritional benefits of fish consumption via NHS sources (“*No one had that conversation with me*” #009). There was routinely a brief discussion with their midwife about which foods should be limited or avoided at the booking appointment, but none received tailored information from a midwife about nutrition, and none talked with their midwife specifically about the benefits of fish as part of a healthy diet (“*If somebody could have said to me this fish would be particularly good for you, why don’t you just try it? I would have given it a go*” #002). Some women did not feel confident either safely preparing or cooking seafood at home (“*I don’t know how to cook them”* #012), and were more likely to order fish when at a restaurant: *“chefs are probably better at cooking it than I am at home”* (#010). Others associated fish with the extra effort of having to make sure *“the bone and skin is off”* (#009). Smell and changes in taste were common, but although most women mentioned aversions to particular food or food groups, nausea, sickness and in one case hyperemesis, few discussed “*smell, taste”* (#006) or had no appetite for certain types of fish: “*eventually I stopped eating raw fish, it was too fishy”* (#015), *“I couldn’t eat sardines and pitta bread for a very long time”* (#001). Only one woman felt they could get *“whatever is in it”* via a supplement (#009). Having a partner who enjoyed cooking and eating fish impacted some women’s fish consumption; in contrast, if the participant was only cooking it for themselves, fish was an *“easy one to cut out”* (#003). Food subscription boxes containing fish were described to be “*at a premium price*” (#010) and the cost of fish was mentioned by a minority of participants, and this was not a main factor when deciding whether or not to consume fish. If a participant particularly liked fish they would buy it from a trusted source, such as local fresh fish markets, where it was a “*bit more expensive*” (#011). One participant discussed the fact that cheaper fish options were available such as canned or frozen, but that these may be overlooked because they were viewed as “*old fashioned*” (#011). PEAR participants were pregnant during the COVID-19 pandemic (most during lockdowns), and one specifically did not want to venture out to busy “*market*” environments where fresh fish were sold (#012). A few women discussed sustainability and the environmental issues relating to their reduced fish consumption (#003, #007, #008). Making *“better lifestyle choices”* (#003) by reducing fish and meat consumption were informed by initiatives such as “*Fish Free February*” (#008) and the 2021 documentary *Seaspiracy* [26], which highlights *“the horrors of fishing”* (#007).

## 4. Discussion

In this mixed-methods study, we found that women who ate fish before pregnancy reduced their intakes of both oily and white fish during pregnancy, with a few choosing to avoid it altogether. Compliance with advice on avoiding shark/marlin/swordfish was high. There was no evidence that women exceeded the limit on tinned tuna, but there was evidence of mis-recall on the suggested limit. However, overall intakes of fish were below that recommended during pregnancy (at least two portions a week, of which at least one—but no more than two—should be oily [2,10]). The barriers to fish consumption in pregnancy included risk aversion, confusion over and mis-recall of the specific details of the guidance, and factors related to cost, availability, family preferences and smell/taste. The NHS website was the main source of information, with midwives also being highly trusted.

The aim of the current guidance on fish consumption in pregnancy in England is based on avoiding or minimising microbiological and toxicological hazards. Specifically, these include: (1) mercury from tuna and high-level predatory fish, (2) pollutants such as dioxins and polychlorinated biphenyls from oily fish, (3) bacteria, viruses and toxins from raw shellfish, and (4) listeria from uncooked smoked fish (update in 2022 after data collection reported here). There is no doubt of the benefit of avoiding microbiological hazards from fish in pregnancy. In 2019, for example, pregnancy-associated cases of listeria accounted for 18% of all cases and one-third of these cases resulted in stillbirth or miscarriage in England [27]. Similarly, mercury is a toxin associated with an increased risk of adverse developmental effects including neurodevelopmental disorders, and is recognised by the World Health Organization as being among the top ten chemical or groups of chemicals of major public health concern [28]. However, there is increasing recognition that fish eaten in pregnancy may have positive effects on the neurodevelopment of the fetus that outweigh adverse effects of mercury, possibly through provision of essential nutrients such as long-chain fatty acids, iodine, vitamin D, selenium and choline [7,9,29]. Thus it is important that NHS advice on fish consumption reflects SACN’s recommendations on limiting or avoiding certain fish while still including the overarching positive message on fish consumption.

Despite the importance of this guidance in assuring the optimal development of the fetus, its effect on pregnant women’s fish consumption in England is unknown. Indeed, there are few data on the consumption of fish in pregnancy, but in accordance with our findings they have shown intakes below those recommended by SACN on which the NHS guidance is based. These include the ALSPAC study in 1991–1992 (40% ate white fish at least once per week, and only 23% ate oily fish 1–3 times per week, with 18% never or rarely eating any white fish, and 43% never or rarely eating any oily fish [30]), the Southampton Women’s Study in 1998–2007 (only 36% ate oily fish once a week or more) [15] and the CARE Study in 2003–2006 (oily fish intakes in pregnant women who were consumers was 101 g/week (<1 portion)) [14]. For all adults, the National Diet and Nutrition Survey (NDNS) found mean intakes of oily fish of 56 g per week (<<1 portion) in 2016–2019 [16]. In the NHANES study in the USA, many women were eating fewer than the USA recommendation of two servings of low-mercury fish a week, but no difference was found in the prevalence of seafood intakes between pregnant and non-pregnant women (1999–2006) [31]. This is the first study to report data on the effect of pregnancy on fish intake in England to our knowledge: the proportion of consumers of white and oily fish both declined (by 3 and 7 percentage points, respectively) and there was evidence of a decline in the frequency of consumption in consumers. While these trends are not large, they are declines from a low baseline consumption and document intakes falling even further below the recommendations during pregnancy.

We used qualitative in-depth interviews to elicit information about the reasons for these changes. Several of the participants reported that they had avoided fish in pregnancy for the health of their baby, and it was common to state that they ‘did not want to take the risk’. Risk aversion has frequently been reported in connection with fish consumption in pregnancy. For example, a national advisory warning on mercury in the USA triggered an unexpected decline in fish consumption in pregnant women [32] and risk aversion was found to permeate multiple themes that shaped Australian women’s perceptions of fish and seafood as part of a healthy diet in pregnancy [13]. This stands in contrast to determinants of fish and seafood consumption in a systematic review of adults, in which the major influences were not risk aversion, but were related to health, environmental influences and personal preferences [33]. Similarly, large proportions of pregnant women in Ireland, Canada and Australia still ate foods that were high-risk for listeria and other foodborne illnesses in contravention of the guidance in those countries [34,35,36].

As well as risk aversion, common themes that formed attitudes to fish consumption in pregnancy included cost, availability, family preferences, lack of confidence in cooking and preparation, and dislike. Conversely, many were aware of the health benefits. Similar themes were identified in a systematic review of perceptions of consuming fish in pregnancy in 2015 [9].

Previous studies have highlighted the problem of lack of awareness of the guidance generally on foods to avoid or limit, particularly among lower socio-economic groups [34,35,36]. In our study (in which participants for the online questionnaire had a greater proportion of households with incomes ≥£50,000 than national indicators) misinformation was evident particularly for some of the details of the guidance, notably for tinned tuna, for which there was widespread lack of knowledge that the limit was four medium-sized cans a week (with the misperception that it was two). In contrast, there was high awareness of and high compliance with the guidance on marlin/shark/swordfish, but this was rarely eaten pre-pregnancy.

Women generally do not receive adequate nutrition education during pregnancy, and although this is thought to be important by healthcare providers, they lack time, resources and relevant training [37]. In this study, women also reported that they felt there was not enough time to have a discussion about nutrition specifically tailored to their particular diet or circumstances. Midwives signposted the availability of information about foods to avoid or limit, including fish on the NHS website. A similar need for information from healthcare providers on fish consumption and clarification on suitable types of fish was identified in Australian women [13].

Health-related behaviours, including those that are nutrition-related, seem to change little during pregnancy, especially in women that have few educational qualifications [38] and pregnancy would appear to be an ideal opportunity to provide guidance on this. The NHS website was identified as the main source of information on foods and drinks to avoid or limit during pregnancy in this study, and it is therefore important that it conveys information in a way that is easily understood and remembered. Most of the messages are negative (loss-framed; i.e., Do not eat …): while this style is generally thought to be effective for women who are well informed, it may not be so effective for less well-informed women [39]. Although most of the participants reported that they found the website clear and accessible, it was clear that some of the information was not retained (for example, the maximum number of cans of tuna per day) and not always understood (for example, advice on smoked salmon).

### 4.1. Strengths and Limitations

We used a mixed-methods design to collect quantitative data from a questionnaire with qualitative data on women’s decisions on fish consumption during pregnancy. Those completing the survey were from all areas of England, but were not completely representative of the population. Those taking part in the qualitative study were even less representative, and it is likely that women with an interest in nutrition were more highly educated. Thus women who avoided fish for reasons of cost and/or availability were less likely to have taken part. However, we have been able to provide empirical data and identify clear themes that have enabled us to provide key recommendations for improvement of the NHS website on guidance on fish consumption in pregnancy.

### 4.2. Conclusions

We found that pregnant women were not eating the recommended amount of fish either before or during pregnancy, and frequently reduced their intakes compared with before pregnancy. The NHS guidance is the main source of information for advice on fish consumption in pregnancy. Although the guidance on the website was thought to be generally clear, there was evidence of mis-recall and confusion, leading to risk-averse behaviour. There are some changes that could be made to improve its effectiveness: (1) The overall message to eat at least two portions of fish a week, one of which (but no more than two) should be oily, should be prominently shown. (2) Tinned tuna is commonly eaten but the message on the number of cans of tuna advised per week is poorly recalled and needs to be disseminated accurately. (3) The advice about shark/marlin/swordfish could receive less prominence as it is rarely eaten either before or during pregnancy. Messages could be presented in a positive way (gain-framed) for maximum effectiveness. Clarity and simplicity of the guidance, with an overall positive message on the number of portions advised per week prominently shown, would help pregnant women to benefit from the nutrients in fish while minimising exposure to pollutants and toxins.

## Figures and Tables

**Table 1:** Questions included in the online questionnaire in relation to fish consumption.

Questions in Relation to Fish	Response Options
Thinking about fish, was there any difference in how often you ate it during your recent pregnancy compared with before you were pregnant?	Ate more often Ate same Ate less often Ate before pregnancy but avoided during recent pregnancy Don’t eat anyway Don’t know/Can’t remember
Before your recent pregnancy how often did you eat fish? While you were pregnant recently how often did you eat fish?	Never Less than twice a week Twice a week More than twice a week Don’t know/Can’t remember
Before your recent pregnancy how often did you eat these types of fish and seafood? While you were pregnant recently how often did you eat these types of fish and seafood?	Never Less than once per month 1–2 times a month Once a week Several times a week Don’t know/Can’t remember
- White fish (e.g., haddock, cod, pollock, tilapia, including breaded fish and fish fingers) - Oily fish (e.g., salmon, trout, mackerel, herring) - Shark/marlin/swordfish - Tinned tuna - Fresh tuna - Shellfish (e.g., mussels, lobster, crab, prawns, scallops, clams)	

**Table 2:** Demographic characteristics of postpartum women who completed the online questionnaire.

Characteristic	Completing Questionnaire			Completing In-Depth Discussion	
	n	Value	National Indicator [23,24,25]	n	Value
Age (years)	548	Range 21–46, Median 33 (IQR 30–36)	Mean maternal age at birth 30.5	14	Range 30–41, Median 34
18–25					0
>25–35					9
>35					5
Home location	598			14	
North East/North West/Yorkshire and Humberside		153 (26%)	28%		0
East Midlands/West Midlands		106 (18%)	20%		3
East/Greater London/South East/South West		339 (57%)	53%		11
Highest educational attainment	596			14	
None/GCSE/Vocational level 1 and 2/AS or A level/Vocational level 3		114 (19%)	50%		1
University degree (BSc, BA)/Professional qualification/Vocational levels 4 and 5/University higher degree (MA, MSc, PhD)		482 (81%)	50%		13
Household income	561			14	
<£30,000		89 (16%)	50%		4
≥£50,000		472 (84%)	50%		10
Parity	597			14	
1		432 (72%)	42%		8
>1		165 (28%)	58%		6
Ethnicity	593			14	
White		563 (95%)	80%		12
Other		30 (5%)	20%		2
Age of baby (months)	598			14	
0–5		371 (62%)			10
6–12		227 (38%)			4
Followed a particular diet before pregnancy	598			14	
Yes		122 (20%)			2
No		476 (80%)			12
Paid work during pregnancy	598			14	
Yes		547 (92%)			11
No		51 (9%)			3
Smoking	596			13	
No		576 (97%)			12
Yes		20 (3%)			1
Home internet access	598			14	
Yes		598 (100%)			14
No		0 (0%)			0

Values are n (%).

**Table 3:** Change in frequency of total fish consumption from before pregnancy to during pregnancy (online questionnaire).

Total Fish	N		Frequency of Consumption in Total Group ^a^					Compliance with Guidance on Total Number of Portions per Week ^b^	
	Total ^a^	Consumers	Never	<Twice per Week	Twice per Week	>Twice per Week	Chi Square Test *p* Value	All Respondents	Consumers Only
Before pregnancy	595	500 (84%)	95 (16%)	319 (54%)	142 (24%)	39 (7%)	<0.001	181 (30%)	181 (36%)
During pregnancy	595	495 (83%)	100 (17%)	338 (57%)	131 (22%)	26 (4%)		157 (26%)	157 (32%)

^a^ Excludes ‘Don’t know/Can’t remember’ (n = 2 for before pregnancy, n = 3 for during pregnancy). ^b^ Guidance in England (Supplementary Table S1) [2,3,5,10]. Non-pregnant women: Eat at least two portions per week, one of which should be oily (Twice a week/>Twice a week). Pregnant women: Eat at least two portions of fish a week, including at least one but no more than two oily (Twice a week/>Twice a week).

**Table 4:** Change in frequency of consumption of types of fish and shellfish from before pregnancy to during pregnancy (online questionnaire).

Fish Type	N		Frequency of Consumption in Total Group ^a^					
	Total	Consumers	Never	Less than Once per Month	About One or Two Times per Month	About Once per Week	Several Times per Week	Chi Square Test *p* Value
White fish								
Before pregnancy	597	483 (81%)	114 (19%)	104 (17%)	246 (41%)	123 (21%)	10 (2%)	<0.001
During pregnancy	598	466 (78%)	131 (22%)	120 (20%)	205 (34%)	133 (22%)	8 (1%)	
Oily fish								
Before pregnancy	595	405 (68%)	190 (32%)	93 (16%)	159 (27%)	131 (22%)	22 (4%)	<0.001
During pregnancy	596	364 (61%)	232 (39%)	87 (15%)	144 (24%)	118 (20%)	15 (3%)	
Shark/marlin/swordfish								
Before pregnancy	586	45 (8%)	541 (92%)	45 (7%)	0 (0%)	0 (0%)	0 (0%)	<0.001
During pregnancy	590	5 (1%)	585 (99%)	5 (1%)	0 (0%)	0 (0%)	0 (0%)	
Tinned tuna								
Before pregnancy	595	420 (71%)	175 (29%)	112 (19%)	182 (31%)	103 (17%)	23 (4%)	<0.001
During pregnancy	593	377 (64%)	216 (36%)	113 (19%)	157 (26%)	95 (16%)	12 (2%)	
Fresh tuna								
Before pregnancy	588	163 (28%)	425 (72%)	131 (22%)	22 (4%)	5 (1%)	5 (1%)	<0.001
During pregnancy	587	50 (9%)	537 (91%)	39 (7%)	11 (2%)	0 (0%)	0 (0%)	
Shellfish								
Before pregnancy	593	359 (61%)	234 (39%)	170 (29%)	135 (23%)	49 (8%)	5 (1%)	<0.001
During pregnancy	595	216 (36%)	379 (64%)	108 (18%)	75 (13%)	30 (5%)	3 (1%)	

For details of guidance in England see Supplementary Table S1 [2,3,5,10]. ^a^ Excludes ‘Don’t know/Can’t remember’ (n = 0 for before pregnancy and n = 0 for during pregnancy for all data except for shellfish before pregnancy n = 2).

## Data Availability

Underlying data are subject to an embargo until the end of the study funding in 2025. The data will then be made available to bona fide researchers on application from data.bris.ac.uk/data (accessed on 11 June 2023).

## Supplementary Materials

The following supporting information can be downloaded at: https://www.mdpi.com/article/10.3390/nu15143217/s1, Table S1: Summary of NHS England guidance (2021) on seafood consumption; Table S2: Themes, sub-themes/codes and illustrative quotations.
