# Supplementary material for: Fish Consumption during Pregnancy in Relation to National Guidance in England in a Mixed-Methods Study: The PEAR Study
Source: Nutrients. 2023 Jul 20;15(14):3217. doi: 10.3390/nu15143217 (PMC10384333; doi:10.3390/nu15143217)
Supplement: Supplementary file 1 [file nutrients-15-03217-s001.zip › nutrients-2474784-supplementary.pdf]

Fish consumption during pregnancy in relation to national guidance in England in a mixed methods study: The PEAR Study (Pregnancy, the Environment And nutRition)

Supplementary Text S1 PEAR interview women's topic guide

TOPICS	PROMPTS
**Background**	
1. How are you/baby/family doing?	How old is baby? Adjusting, siblings, maternity leave. How much support do they have at present? Do you have a partner living with you? Issues relating to pandemic?
2. How did you find out about this research study?	Where did you see the study advert? / Who told you about it?
3. What did you think about the questions we asked in the questionnaire?	Any that stood out as particularly relevant to them, not relevant?
**Nutrition information received**	
4. During your recent pregnancy did you get any particular information about food and diet from a healthcare provider?	For example, a midwife, health visitor or GP?<br>How many weeks? Any reminders later on?
5. When is a good time to have these conversations (booking appt?)	
6. Did you get information from other sources?	Website, apps, magazine, other people, sent information in post prior to booking appt?
**Dietary changes**	
7. Did you make any changes to your diet when you found out you were pregnant?	Why? (nausea, cravings, advised to eat more or less of a food item, wanted to be healthier)
8. Did your eating patterns change as your pregnancy progressed?	How did your diet change? When? / Why (nausea, cravings, advised to eat more or less of a food item)?
**Perceptions of fish and seafood**	
Women who are pregnant make changes to their diets for lots of different reasons, but one type of food we are especially interest in is fish and seafood.	**Establish if they ate less/same or more fish during recent pregnancy**
9. When you think about fish and seafood, what comes to mind?	
10. Did you eat fish before you were pregnant?<br>11. Did you still want to eat fish when you found out you were pregnant?	(e.g. Oily fish (e.g. salmon, trout, mackerel, herring) Tinned tuna, fresh tuna, while fish (e.g. haddock, cod, pollock, tilapia, including breaded fish and fish fingers) Shark, marlin or swordfish)

12. *If fish eaten, if not go to 16:* What types of fish did you eat during your pregnancy? (Same/different?)	What seafood did you eat?<br>Shellfish<br>(e.g. mussels, lobster, crab, prawns, scallops, clams)<br>Cost, availability in supermarket, eating out in restaurants?<br>How do you eat it?<br><br>**Establish reasons for eating more or less fish during pregnancy**
13. Can you tell me what influenced your choice of types of fish to buy when you were pregnant? (Same/different from fish prior to pregnancy?)<br>14. If yes, how do you buy it fresh, frozen, prepared?	price, availability, prep and cooking knowledge, smell, bones, whether family like it, eating out in restaurants?<br>Establish if this is any different to the way in which it was prepared/bought prior to pregnancy?
15. Did you change your fish/seafood intake because of guidance / advice?	Clearer information? Information in a different format? Recipe suggestions? Info about prep? Reminders from health professionals? Who?
16. *If not eaten or fish intake reduced:* What do you think might have helped you eat fish regularly during your pregnancy?	Health aspects (positive and negative), price, availability, prep and cooking knowledge, smell, bones, whether family like it
17. What have you heard about the health effects of eating fish during pregnancy?	Where did they hear about this?<br>Do they trust this information?
**Perceptions of NHS Choice/FSA website information**	**Prompt: open links to websites**
**Ask participant to open: NHS link**<br>The government/NHS provide advice on eating fish during pregnancy<br>Explore if/how they hear about it…<br>**For NHS Choices fish advice:**	
18. Have you seen this information before?	Did you look for it off your own back?
19. What is the key message you get from the information?	Does it make them feel scared of eating fish/seafood?

20. How does the information make you feel about eating fish and seafood?<br><br>21. Did this information help you make decisions about eating fish/seafood during your recent pregnancy?<br><br>22. Are there any other ways that you think this information could be provided that would have been more helpful for pregnant women? [e.g. in an App, leaflet, or designed differently]	Do they find this information confusing/straight forward or sensible?<br><br>Is it alarming, does guidance put them off eating fish all together?<br><br>Would they have valued a discussion with their midwife about this?<br>If yes, **when would be the best time to have this discussion?**<br><br>**Important:** Emailed message 'have you had at least 2 portions of fish' this wk. Start for life email at 6 weeks - Was it something I ate?<br><br>what is oily / white fish – pictorial, interactive<br><br>USA hand with portion sizes),<br><br>more discussion with midwife<br><br>**When would you like the information?**
**Final questions …**	
23. Is there anything you wish you had been told or that someone had discussed with you about diet during your pregnancy?<br><br>24. Do you have any comments or thoughts about what we have discussed today?<br><br>25. Do you have any questions arising from the information [links]?	Provide clarification where possible or direct to website/PI who can provide further information where needed.

This is a guide only, adjustments to the topic guide will be documented/made if necessary. Interviewer to use further prompts to explore areas of interest as they arise during the discussion. Interviewer to use own discretion to omit questions or alter wording as appropriate during interviews.

Supplementary Table S1 Summary of NHS England guidance (2021) on seafood consumption[1-3]

Seafood item	Hazard	General population	Specific population group				
			Women of childbearing age	Planning a pregnancy	Pregnant	Breastfeeding	Children
Fish[a]		Eat at least two portions per week, one of which should be oily	-	-	-	-	-
Oily fish[ab]	Toxins: mercury, dioxins and polychlorinated biphenyls	At least one portion a week but no more than two portions a week (women) or four portions (men)	At least one portion a week but no more than two portions per week	At least one portion a week but no more than two portions per week	At least one portion a week but no more than two portions per week	At least one portion a week but no more than two portions per week	Girls: At least one portion a week but no more than two portions per week<br>Boys: At least one portion a week but no more than four portions per week
White fish[a]	Toxins	Unlimited but sea bream, sea bass, turbot, halibut and rock salmon should be avoided by people who regularly eat a lot of fish	-	-	-	-	-
Shark, marlin, swordfish[ab]	Toxin: mercury	No more than one portion a week		Avoid	Avoid	No more than one portion a week	Avoid
Tuna[ab]	Toxin: mercury	-	-	No more than 2 tuna steaks per week (140 g per portion) or 4 medium-sized cans (140 g) of tuna (tuna does not count as an oily fish)	No more than 2 tuna steaks per week (140 g per portion) or 4 medium-sized cans (140 g) of tuna (tuna does not count as an oily fish)	No limit	-
Shellfish[ab]	Microbiological	No limit (except for brown crab recommendation)	-	-	Avoid uncooked shellfish	-	Avoid uncooked shellfish
Brown crab meat[a]	Toxins	Anyone who regularly eats a lot of fish should avoid	-	-	-	-	-
Raw fish[b]	Microbiological	-	-	-	Avoid raw or lightly cooked fish in sushi unless the fish has been frozen first [c]	-	-
Smoked fish (e.g. salmon, trout)[b]	-				Acceptable within oily fish limits [d]		
Fish liver oil supplements[a e]	Teratogenesis: vitamin A	-	-	Avoid	Avoid	-	-

Table shows guidance at April 2021.
Reasons for avoidance/limiting fish are stated as [2]:

- You should limit tuna because it has more mercury in it than other fish. If you eat too much mercury, it can be harmful to your unborn baby.
- You should limit oily fish because they can have pollutants such as dioxins and polychlorinated biphenyls in them. If you eat too much of these, they can be harmful to your unborn baby.
- You should avoid raw shellfish because they can have harmful bacteria, viruses or toxins in them. These can make you unwell and give you food poisoning.

[a]NHS (2018) Fish and shellfish: Eat well. https://www.nhs.uk/live-well/eat-well/fish-and-shellfish-nutrition/ [1]
[b]NHS (2018) Foods to avoid in pregnancy. https://www.nhs.uk/conditions/pregnancy-and-baby/foods-to-avoid-pregnant/ [2]
[c]Advice on sushi was revised in early 2022 to include eating only sushi that comprise cooked fish.
[d]Advice on smoked fish was revised in early 2022 to include thorough cooking of smoked fish after a listeria outbreak linked to smoked fish.
[e]NHS (2020) Vitamins, supplements and nutrition in pregnancy. https://www.nhs.uk/pregnancy/keeping-well/vitamins-supplements-and-nutrition/ [3]

Supplementary Table 2 Themes, sub-themes/codes and illustrative quotations

Themes	Sub-themes/codes	Illustrative quotations
**1. Changes in fish consumption during pregnancy [N = 14]**	**Ate less fish [6]**<br>For the health of baby [5]<br>Effort to remember the number of portions consumed in a week [5]<br>Had no appetite for it [1]	*So I remember thinking well actually maybe those things [seafood] are best limited or avoided entirely just because it seemed easier than trying to keep track of how many portions of something I'd had in a week. #006 1st time mum - Ate fish less often during pregnancy*<br>*I don't want to even take that risk, I just don't want to… #005 Multip - Ate fish less often during pregnancy*<br>*I used to eat a lot of smoked salmon as well. I wasn't aware that you could eat smoked salmon when you were pregnant, I always thought you couldn't, but I don't know if that's right, … it's easier to just avoid it completely rather than get it wrong. #003 Multip - Ate fish less often during pregnancy*<br>*I think because you need to limit the oily fish I felt like that I can't remember how many I have had this week so I might just not have any. #008 1st time mum - Ate fish less often during pregnancy*<br>*…all the food[s] that I enjoy eating are the ones that you're not meant to eat, so I love tuna, I know you can eat canned tuna, but I made sure I limited that, I only had one can a week…It's a difficult though isn't it, because seafood is really good for you isn't it? And it's really good for the baby… #005 - Multip - Ate fish less often during pregnancy*<br>*So I just made sure to only eat salmon once a week so that in case I wanted fish another time I could… It was because I knew that there were certain types of fish you weren't supposed to get a lot of and I just couldn't really be bothered to remember what they were. #015 - Multip - Ate fish less often during pregnancy*<br>*I think I worried more that I was having too much rather than you should still have your oily fish. So I think I would have cut down quite a bit on it … #014 - Multip - Ate fish less often during pregnancy*

		it was thinking maybe in the back of my mind I know I had read about limiting seafood in pregnancy, but also it was mainly just that I didn't fancy it. #006 1st time mum - Ate fish less often during pregnancy
	**Ate the same amount of fish [4]**<br>More awareness around fish consumption [2]<br>Don't eat a lot of fish [1]<br>Don't eat a lot of fish that should be avoided [1]<br>An awareness that oily fish consumption was higher than two portions per week [1]	*… just being more conscious of how and what [fish] I am eating. #011 Multip, 35yrs - Ate same amount of fish during pregnancy*<br>*I know that there's some things that you could still eat [tinned tuna & salmon] but not too much of because of the levels of whatever is in it, so I was aware of that. #009 - 1st time mum - Ate same amount of fish during pregnancy*<br>*the main things to avoid were probably things I wouldn't really have eaten much of anyway…[e.g. tuna] #010 1st time mum - Ate same amount of fish during pregnancy*<br>*I think with knowing that it was a healthy option with fish oils, but also knowing that I was ignoring the fact that I shouldn't be eating more than two portions of oily fish a week… #001 - 1st time mum - Ate same amount of fish during pregnancy*<br>*… probably didn't change the amount of fish I ate at all really, because I ate it so little before it was never anything I had to worry about cutting back on in the first place. #009 - 1st time mum - Ate same amount of fish during pregnancy*
	**Ate more fish [3]**<br>Due to gestational diabetes [1]<br>Tuna didn't make me feel sick [1]<br>Fish is a healthy thing to eat [1]	*I think mine went up once I had been diagnosed with gestational diabetes, because it was something that I didn't try too much after throwing it up in the early days of pregnancy, but once I was diagnosed with GD and I was like well I can't continue with the diet that I was having, it's going to damage my baby #004 - 1st time mum - Ate fish more often during pregnancy*<br>*I didn't reduce my intake of tuna because I don't eat it very often, but because it was one of the few things that didn't make me feel sick… I was probably eating more of it [tuna] than I was before…I was aware that I couldn't eat very much of it. #002 - 1st time mum - Ate fish more often during pregnancy*

		we might have had salmon one day and a white fish another day, and a bit more fish than we were eating [prior to pregnancy]. it's quite a healthy thing to eat. #012 - Multip - Ate fish more often during pregnancy
	**Don't eat fish: Vegetarian [1]**	*I am a vegetarian so the meat and the fish thing is quite simple for me, I don't eat it anyway… I am quite anti-fish now because of the save the ocean. #007 - 1st time mum – Vegetarian/no fish*
	**Fish eaten and enjoyed during pregnancy [13]**<br>Oily fish (salmon) [11]<br>Tuna (tinned) [10]<br>Shellfish (prawns, scampi, mussels) [6]<br>White fish [5]<br>Fish and chips [3]<br>Sushi (with cooked fish) [2]<br>Sushi/poke (Japanese/Hawaiian with raw fish) [1]<br>Oily fish: mackerel, sardines [1]	*The stuff that I would eat I would literally eat tinned tuna, tinned salmon, scampi…. #009 - 1st time mum - Ate same amount of fish during pregnancy*<br>*I had a really strange craving for a nice prawn sandwich, #010 1st time mum - Ate same amount of fish during pregnancy*<br>*…I would eat white fish, I would eat cod definitely, I had quite a lot of cod. I had tuna about once a week. I ate smoked salmon a lot. #005 - Multip - Ate fish less often during pregnancy*<br>*I guess we're looking at fish being a health thing, but obviously I think this being England a lot of people's fish your first stop would be fish and chips, but I suppose that's not necessarily a health food, I don't know. I probably had fish and chips a few times through the pregnancy, but not excessively so. #008 1st time mum - Ate fish less often during pregnancy*<br>*I tried to have ones [sushi] with say cooked tuna, cooked prawns, vegetarian ones. #008 1st time mum - Ate fish less often during pregnancy*<br>*I ate sushi, obviously we're limited in that it's expensive. A couple of times I got [supermarket] sushi which even I would admit was not the wisest choice in terms of how well prepared that sushi is. So again I limited it. I ate poke for a while… #015 - Multip - Ate fish less often during pregnancy*<br>*we'd have sardines, quite a lot of tinned sardines, quite a lot until I was nauseas, we had tinned tuna and salmon, quite a lot of smoked mackerel, like the fresh smoked mackerel,*

		and then white fish, sea bass, fish cakes, haddock, just any fish really that was going. #001 1st time mum - Ate same amount of fish during pregnancy
**2. Consistent fish messages: Avoid, limit, and cook thoroughly [14]**	**Eat in moderation [8]**	*So I knew that the tinned tuna and salmon I could eat but in moderation #009 - 1st time mum - Ate same amount of fish during pregnancy*<br><br>*[key message from NHS fish guidance] I think it's moderation… .#010 1st time mum - Ate same amount of fish during pregnancy*<br><br>*you're okay to eat it [smoked salmon] in moderation. #014 - Multip - Ate fish less often during pregnancy*<br><br>*we have prawns quite often but cut that down #011 Multip - Ate same amount of fish during pregnancy*<br><br>*there's all those things about not too much tuna #012 Multip - Ate fish more often during pregnancy*<br><br>*…don't eat too much seafood like marlin or tuna or whatever. #007 1st time mum - Vegetarian with no fish*
	**Avoid shark, marlin and swordfish [10]**<br><br>Shark [5]<br><br>Marlin [4]<br><br>Swordfish [4]<br><br>Information on outside of plastic folder [3]	*I had a plastic wallet to put our notes in, and on the front of the wallet it had a list of what to avoid, but we [midwife] never had that discussion, we never spoke about it. It was just a list of what to avoid eating. #005 - Multip - Ate fish less often during pregnancy*<br><br>*… on the outside of the plastic folder it said to avoid shark and marlin, and raw eggs, and seafood ... I don't even know where you buy shark from, but I suppose if you were on holiday somewhere just to be aware that this could be in some restaurant food … #002 - 1st time mum - Ate fish more often during*<br><br>*"we were actually [on holiday] and we had marlin" #014 - Multip - Ate fish less often during pregnancy*<br><br>*pregnancy I remember swordfish, I don't know why, and shark, just because it seems unusual in a British diet. #006 - 1st time mum - Ate fish less often during pregnancy*

		here's a few things that you're probably not likely to eat anyway, how many people eat shark on a day to day basis? #010 1st time mum - Ate same amount of fish during pregnancy<br><br>*I am sure my midwife asked me if I eat swordfish and I said no, and she just said to continue to avoid it whilst pregnant. #003 - Multip - Ate fish less often during pregnancy*
	**Limit tuna [11]**<br><br>Limit tuna because of mercury [4]<br><br>Avoid, limit certain fish because of mercury [2]	*Limiting tuna, the sea fish like marlin, not that I eat that… #011 Multip, 35yrs - Ate same amount of fish during pregnancy*<br><br>*I didn't get tuna if I got sushi for the most part #015 - Multip - Ate fish less often during pregnancy*<br><br>*I don't like tuna steaks, but I like tuna in a can. But I remember reading about it, and that you have got to limit some of them…#009 - 1st time mum - Ate same amount of fish during pregnancy*<br><br>*I know I could have had a tin [of tuna], but I avoided it quite a lot, because it's probably one of the ones that most of the time they tell you to avoid don't they? #014 - Multip - Ate fish less often during pregnancy*<br><br>*the salmon and the tuna were the ones that I think I had to think about more and how much I eat of it during pregnancy. WM003 - Multip - Ate fish less often during pregnancy*<br><br>*I remember reading about the metallic elements in seafood, and things like mercury build-up and that actually that's why you should limit things like tuna steaks and other types of fish. #006 - 1st time mum - Ate fish less often during pregnancy*<br><br>*[avoid] the fish with the higher metal content… I eat a bit of tuna #010 1st time mum - Ate same amount of fish during pregnancy*

		we don't eat tuna anyway necessarily, tuna fish from a tin maybe, and I know things like what is it? They're worried about ones that might have mercury in them #012 Multip - Ate fish more often during pregnancy<br><br>*I know you're not supposed to eat a lot of mercury basically #015 - Multip - Ate fish less often during pregnancy*<br><br>*I was really interested that there are certain types of fish you shouldn't eat because of the mercury levels #007 1st time mum - Vegetarian with no fish*
	**Limit salmon [11]**<br><br>**Limit oily fish [8]**<br><br>Omega3, oily fish - health benefits [3]	*I know that salmon is good but in small doses isn't it? #003 - Multip, 30yrs - Ate fish less often during pregnancy*<br><br>*…if I was going to have salmon on it [sushi] I would have limited how much I ate. #014 - Multip - Ate fish less often during pregnancy*<br><br>*I remember there was something about oily fish, and that was the only thing that I ate more of that I remember being told not to eat more than two portions or something, and that's the only thing I can remember. #001 1st time mum - Ate same amount of fish during pregnancy*<br><br>*I did take a pregnancy vitamin and there were omega 3 fish oils in it, but no I didn't have any other link to it being particularly healthy or not. #001 1st time mum - Ate same amount of fish during pregnancy*<br><br>*I feel like there's fatty acids, omega 3, whatever it is that's in salmon, and I'm not sure what else it's in, that's promoted as a good thing. I couldn't tell you where I heard that from. #003 - Multip, 30yrs - Ate fish less often during pregnancy*<br><br>*I did think I wonder whether I should try and stomach some oily fish, just because as I say I know omega 3 and 6 is good for brain development #006 1st time mum - Ate fish less often during pregnancy*
	**Avoid raw, rare fish/shellfish [9]**	*we didn't have raw fish… #008 1st time mum - Ate fish less often during pregnancy*

		...I definitely remember avoiding sushi. #001 1st time mum - Ate same amount of fish during pregnancy<br><br>*In terms of fish I could eat a salmon fillet generally but it had to be cooked thoroughly the whole way through, it would have had to have been anyway because I was pregnant. #004 1st time mum - Ate fish more often during pregnancy*<br><br>*people would say to me you can't eat prawns or something, and I felt by being on the NHS website I could say no it quite clearly says on the NHS website you can have it…the raw shellfish I would have avoided…I like eating sushi but I would also make sure it wasn't raw… #014 - Multip - Ate fish less often during pregnancy*
**3. Fish guidance is the most complicated [12]**	**How many portions a week? [10]**<br><br>Two portions a week – incorrectly applied to tuna [4]<br><br>I ate one portion per week [salmon/tuna] [4]<br><br>Four medium sized cans of tuna - correctly recalled [2]<br><br>Four cans of tuna - I could have consumed more [3]<br><br>Two portions a week - applied as blanket rule to all fish [4]<br><br>What is a portion? [3]	*It does feel quite complicated. I think the fish one is the most complicated out of all of the ones that I have read because some of these fish are okay, some of them aren't, some of them you have never heard of so why would you be thinking of them? #012 Multip - Ate fish more often during pregnancy*<br><br>*I feel like fish is the only one where there's a limit rather than an avoidance, I can't remember, I don't know. #001 1st time mum - Ate same amount of fish during pregnancy*<br><br>*I did think I could have had more of that [tuna] except I can't because it's advised not to have more than a couple of tins a week. #002 - 1st time mum - Ate fish more often during pregnancy*<br><br>*I think it was this pregnancy where I maybe said ideal lunch is a jacket potato with tuna mayo or something, and it was like, "That's good as long as you don't eat too much of it, how much do you eat in a week?" And it's about a tin, and she said, "I wouldn't eat more than two tins a week. #003 - Multip, 30yrs - Ate fish less often during pregnancy*<br><br>*I love tuna … I eat quite a lot of tuna, so I remember reading that. I think it was two cans a week or something like that #005 Multip - Ate fish less often during pregnancy*

		…tinned tuna was another thing that I ate quite regularly … I wasn't over eating it where I had to take it down for pregnancy… I would only eat up to one tin a week… #003 Multip - Ate fish less often during pregnancy<br><br>*I eat quite a lot of salmon and things like that. But I would always make sure I only had one portion a week, or the thing with the tuna the same. #014 - Multip - Ate fish less often during pregnancy*<br><br>*…during the pregnancy we just made a point in only making it [salmon] once a week. #015 - Multip - Ate fish less often during pregnancy*<br><br>*four medium sized cans of tuna, I do remember that, because I would never eat four cans of tuna a week #001 1st time mum - Ate same amount of fish during pregnancy*<br><br>*you should eat no more than two tuna steaks or four medium sized cans of tuna. It's funny that did stick in my brain because I don't actually eat tuna at all #006 1st time mum - Ate fish less often during pregnancy*<br><br>*Oh wow, [four cans of tuna] a week, yeah, see I would have thought it was just one #014 - Multip - Ate fish less often during pregnancy #014 - Multip - Ate fish less often during pregnancy …*<br><br>*I can actually eat more [tuna] than I thought. … #003 Multip - Ate fish less often during pregnancy*<br><br>*I am sure I remember they [midwife] said something about two portions [of fish] a week seemed an acceptable level that was okay. #006 1st time mum - Ate fish less often during pregnancy*<br><br>*I would have thought it was two portions of fish [per week]… I like fish but I wouldn't have eaten it every day during pregnancy because I would worry there's too much fish. #014 - Multip - Ate fish less often during pregnancy*

		I basically just I would always look it up and I would always forget, and would just be like you just shouldn't eat fish more than twice a week… #015 - Multip - Ate fish less often during pregnancy<br><br>*salmon is the one we eat most of, so whatever the NHS website says about that was what we were doing, but we might have had salmon one day and a white fish another day #012 Multip - Ate fish more often during pregnancy*<br><br>*I suppose two portions is quite subjective anyway, I think that's probably why I didn't stick to it. If it had given me grams or… but two portions I thought well that's really variable anyway. I think I would have followed it more if it had been really evidence based as to exactly how much I could and couldn't eat, but I think because it was just two portions I thought oh well… #001 - 1st time mum - Ate same amount of fish during pregnancy*<br><br>*And also what's a portion... if you bought a packet of smoked mackerel does it say on the packet this counts as two portions? Because you have got portions nutritionally and then portions in terms of what they are allowing you to eat for the safety thing, and I am assuming that's the same things, but not necessarily… #008 1st time mum - Ate fish less often during pregnancy*<br><br>*… I even would look up what would class as a portion, know roughly what size a portion would be, because I liked a lot of salmon, and so I would make sure if I'd had... say if I had smoked salmon for breakfast I would only have maybe a little bit of it, so I would say well I've got 100 gram pack here, that's okay to eat over the week, I can have that because that classes as my two portions or something. #014 Multip - Ate fish less often during pregnancy*
	**Can I eat smoked salmon? [6]**	*I think there was some points were confusing like smoked salmon you can eat, but salmon I think is one of those that you should only have a couple of times a week, so*

		it's like okay well do I have to limit smoked salmon intake too? #004 1st time mum - Ate fish more often during pregnancy<br><br>*But perhaps some of the types of fish that I found, there was some grey areas around things like smoked salmon…It's maybe where I got a bit confused, because cured meats aren't safe but cured or smoked fish is okay, so that's… because they go through quite similar processes, effectively air drying isn't it? So I suppose it's a little bit contradictory.#010 1st time mum - Ate same amount of fish during pregnancy*<br><br>*in my first pregnancy it was told you shouldn't eat smoked salmon, and I was always really confused on smoked salmon whether I should eat it or whether I shouldn't eat it, and then when I researched it again when I was pregnant with [child] it said it was fine, and you're okay to eat it in moderation. #014 Multip - Ate fish less often during pregnancy*
	**If frozen certain fish is okay. Has it been frozen? [4]**	*the one thing I had a little bit of confusion over was freezing… if the fish had been frozen before being used to make sushi for example, because you then have it… which I think the answers were probably yes that I could have it, but again always almost a bit of caution, I think I tended to avoid it #008 1st time mum - Ate fish less often during pregnancy*<br><br>*if the fish has been frozen first, yeah I think I probably wasn't sure, because we weren't making it at home, whether the fish had bene frozen, so I just remember avoiding it. #001 1st time mum - Ate same amount of fish during pregnancy*
	**Can I eat shellfish? [3]**	*I still can't really remember whether I am allowed to eat prawns and mussels and shellfish. I think I would definitely have looked that up to see which fish I can eat or not eat…. #001 1st time mum - Ate same amount of fish during pregnancy*

		I probably wouldn't have given prawns or anything a go, but I am pretty sure you're not supposed to eat prawns anyway. #002 - 1st time mum - Ate fish more often during pregnancy<br><br>*the only other fish that I really eat is mussels and prawns, which again I don't know whether you're supposed to eat those when you're pregnant or not #003 Multip - Ate fish less often during pregnancy*
**4. Reasons for low fish consumption during pregnancy [14]**	**Weighing up risk and benefit: Err on the side of caution [13]**<br><br>Food poisoning and risk [8]<br><br>Trust or lack of - in whether fish is cooked properly [4]<br><br>Consequences of food poisoning [2]<br><br>Lack of messages about benefits of eating fish [3]<br><br>Buying fish from reliable sources [2]<br><br>Lack of messages about benefits of eating fish [9]<br><br>Try to eat at least two portions of fish a week – not widely known [6]	*I used to eat a fair bit of smoked mackerel, which is oily fish isn't it? But I haven't had that, I think I just cut that out actually really in pregnancy, just rather be safe than sorry. I would have had some salmon. #008 1st time mum - Ate fish less often during pregnancy*<br><br>*[I] wouldn't eat cockles or prawns or mussels or anything like that… #005 - Multip - Ate fish less often during pregnancy*<br><br>*one of my friends owns cockle sheds, because we live quite near the coast, and at the end of the week he will give us what he hasn't managed to sell, so we will have it about once a week… and I remember my husband saying, "The prawns I can cook them then that will be fine, I can cook it with some spaghetti," but I still wouldn't do it, I was like no I don't want to even take that risk, I just don't want to… if I can eat something else then I will just eat something else #005 Multip - Ate fish less often during pregnancy*<br><br>*I would want to know well what are my odds, what is food poisoning going to do? Is it a situation where every time I eat an oyster there's a 50/50 chance of a miscarriage? Or is it a situation where it's just like well you might get food poisoning and that will be really it, and the odds are one in 100? That was always the thing I wanted to know. #015 Multip - Ate fish less often during pregnancy*<br><br>*I feel like saying fish is quite important to eat but you need to be careful which fish that you're eating, and I guess but it is saying that shellfish is actually fine usually as long as you cook it properly. My gut feeling about that has always been that I*

		don't trust that it [shellfish] will be cooked properly #012 Multip - Ate fish more often during pregnancy<br><br>*I would just have cooked salmon rather than smoked salmon… #011 Multip - Ate same amount of fish during pregnancy*<br><br>*shellfish are alright as long as it's cooked properly… #010 1st time mum - Ate same amount of fish during pregnancy*<br><br>*Prawns and things it's just about reliable sources. So I wouldn't buy a prawn sandwich out of a vending machine kind of thing, making sure that it was as fresh as possible #011 Multip - Ate same amount of fish during pregnancy*<br><br>*If somebody could have said to me this fish would be particularly good for you why don't you just try it? I would have given it a go… But if there was another type of not too fishy fish then yeah I would have tried it. There was no positive diet information, there was a couple of don't eat, but there was no you really should be eating this #002 - 1st time mum - Ate fish more often during pregnancy*<br><br>*[One of the messages is try to eat at least two portions of fish a week] No one had that conversation with me, and even looking on here under their fish section it doesn't say that you should eat two a week #009 1st time mum - Ate same amount of fish during pregnancy*<br><br>*I liked things like mussels or calamari or things like that, but I was a lot more worried about having that whilst I was pregnant, which I believe from reading research they said you could, but it just worried me being the type of food it is that it would have been dangerous in other ways. #014 Multip - Ate fish less often during pregnancy*<br><br>*that [cooked shellfish] was something that was happy to risk it with. #010 1st time mum - Ate same amount of fish during pregnancy*<br><br>*It made me feel [NHS website] like I would rather just stay away from it [fish/seafood] mostly, which is funny because I know actually the benefits of eating oily fish for brain development and things like that, but I think my pregnant brain was like no these things are best avoided. #006 - 1st time mum - Ate fish less often during pregnancy*

		most people that I know who have had really bad food poisoning over something has been fish or shellfish particularly, and I don't know whether that's true or not, that's a gut feeling about that. So I would have avoided those anyway. #012 Multip - Ate fish more often during pregnancy<br><br>*[did you hear about any health effects of eating fish during pregnancy?] Health benefits? Not especially, no. Only in that I did take a pregnancy vitamin and there were omega 3 fish oils in it, but no I didn't have any other link to it being particularly healthy or not. #001 1st time mum - Ate same amount of fish during pregnancy*<br><br>*[it's not limit to two portions of fish a week, you can eat more than two portions, is that something you were aware of…] No, I would have thought it was two portions of fish. #014 - Multip - Ate fish less often during pregnancy*<br><br>*why people can't just say these are the things you have got to avoid, X, Y, Z, everything else you will probably be alright with, or it's just in case you get food poisoning or something, that would be helpful. #004 1st time mum - Ate fish more often during pregnancy*
	**Lack of tailored information about fish [11]**	*I didn't see anything when you were looking on there for what not to eat, I don't remember seeing more on the emphasis of what to eat. I know that overall oily fish is very healthy and important, especially for is it brain development? I can't remember where specifically I have heard that though. #008 1st time mum - Ate fish less often during pregnancy*<br><br>*I don't really recall having in-depth discussions on diet too much. I think she might have run over the basic things, which are pretty much on that folder anyway, don't eat too much seafood like marlin or tuna or whatever. #007 1st time mum - Vegetarian with no fish*
	**Fish preparation [4]**<br><br>More likely to eat fish in a restaurant [3]	*as somebody who is doing the cooking I have always been a bit nervous of quite a lot of seafood things because actually I don't know how to cook them, so I suppose*

	Lack of confidence - in cooking fish or seafood at home [2]<br><br>Extra effort - Don't like bones, skin on fish [2]	*we wouldn't eat lots of those anyway. My husband likes oysters, but I would never prepare oysters in the house. #012 Multip - Ate fish more often during pregnancy*<br><br>*fish is probably something I quite like to order when I go out for a nice meal, because I think chefs are probably better at cooking it than I am at home, or can do more interesting things with it, and because of lockdown, so environmental factors I wasn't going out to eat #010 1st time mum - Ate same amount of fish during pregnancy*<br><br>*I am quite lazy making a salmon sandwich I've got to make sure the bone and skin is off, so unless I really fancied it I would be like I really can't be bothered to go through the hassle… #009 1st time mum - Ate same amount of fish during pregnancy*<br><br>*My preference is to eat fish that's already filleted, and I'm not a whole fish eating person, I don't like bones in my fish, so we mostly stay away from that anyway #012 Multip - Ate fish more often during pregnancy*
	**Nausea & food aversions [12]**<br><br>Nausea or had no appetite for fish [3]	*I think it was smell, it was taste… #006 1st time mum - Ate fish less often during pregnancy*<br><br>*eventually I stopped eating raw fish, it was too fishy and I couldn't. #015 - Multip - Ate fish less often during pregnancy*<br><br>*often I would eat sardines and pitta bread [prior to pregnancy], but I think I was just feeling nauseas at the time, and I couldn't eat sardines and pitta bread for a very long time, and I've only really just got back into it. . #001 1st time mum - Ate same amount of fish during pregnancy*
	**Partners influence [8]**	*he [partner] likes seafood a lot, so he will tend to try to cook it every opportunity. We eat a lot of prawns and squid, and we don't buy a lot of shellfish but actually … so he does like that kind of thing, and he probably liked it more than me, but he cooks it and we both eat it. #008 1st time mum - Ate fish less often during pregnancy*

		I was just trying to cut down on meat and fish in general for better lifestyle choices, better for the environment… fish was an easy one because I would only be cooking it for myself anyway, so that was the easy one to cut out... #003 Multip - Ate fish less often during pregnancy
	**Didn't eat much fish prior to pregnancy [2]**	*I don't really like fish. I manage the odd fish and chips if there's enough stuff that you can't really taste the fish, and tuna. #002 - 1st time mum - Ate fish more often during pregnancy*<br><br>*The stuff that I would eat I would literally eat tinned tuna, tinned salmon, scampi, and I really fancy that fancy smoked salmon that you get in an afternoon tea sandwich, but that's pretty much it, the rest of it I don't like at all. #009 1st time mum - Ate same amount of fish during pregnancy*
	**Supplements can substitute fish [1]**	*I just think whatever is in It [fish] at the end of the day I could get it as a supplement #009 1st time mum - Ate same amount of fish during pregnancy*
	**Fish can be difficult to source [5]**<br><br>Difficult to source during COVID-19 pandemic [1]<br><br>Food subscription boxes - never a lot of fish [1]	*My husband and I have done food subscription boxes for a few years now… but there's never an awful lot of fish in those, or they tend to be at a premium price, so they probably tend to be either we go for vegetarian things or things with meat in rather than necessarily a lot of fish in them. #010 1st time mum - Ate same amount of fish during*<br><br>*We don't have a fishmonger. We do have a Saturday food market… and particularly with COVID the market did stay open but it wasn't somewhere we felt like… and it wasn't somewhere I wanted to go because I was pregnant so I didn't want to be around lots of people anyway really at that early stage. #012 Multip - Ate fish more often during pregnancy*
	**Sustainability and environmental reasons [3]**	*[neighbour] he's got an initiative called Fish Free February, and you encourage*

		people to cut back on the amount of fish that eat to look at the provenance of the fish #008 1st time mum - Ate fish less often during pregnancy<br><br>*I am quite anti-fish now because of the save the ocean… sustainability… and the horrors of fishing... [partner] recently converted away from fish following Seaspiracy… #007 1st time mum - Vegetarian with no fish*
	**Expense [6]**<br>Cost not an issue [3]<br>Cost a factor [2]<br>Buy tinned/frozen if I can't afford fresh [1]	*I love tuna steaks, they are expensive so not that often. Yes, any time I can get them absolutely, but they aren't featuring often in our diet. #015 Multip - Ate fish less often during pregnancy*<br><br>*we would go and buy king prawns from the fish stall on the market, getting them like that, it was a little bit more expensive than the supermarket but we knew that they were really fresh #011 Multip - Ate same amount of fish during pregnancy*<br><br>*I think people don't think about things like tinned mackerel, because it's a bit old fashioned isn't it? …I think if it [NHS guidance] makes suggestions, where it says oily fish giving ideas of a cheaper option and saying a cheaper alternative such as tinned mackerel or whatever is a good alternative. #011 Multip - Ate same amount of fish during pregnancy*
	**Past pregnancies influence current advice seeking behaviour [3]**	*I think this [NHS guidance] is something that you could go through right at the very start, and just not take for granted if someone has had a pregnancy that they know it. I think that's the biggest thing that's fallen short in what I know, is that I probably have been told much of this almost five years ago, and I really don't remember it now. #003 Multip - Ate fish less often during pregnancy*<br><br>*I thought it was literally one tin of tuna a week. I think it's from my first pregnancy that I thought it was just one, and then I just didn't look it up again… because I didn't know that thing about tuna, and I probably would have eaten more tuna in my pregnancy then, because a good source of protein #014 - Multip - Ate fish less often during pregnancy*

Supplementary references

1. NHS. Fish and shellfish: Eat well. Available online: https://www.nhs.uk/live-well/eat-well/fish-and-shellfish-nutrition/ (accessed on 23 March 2023).
2. NHS. Foods to avoid in pregnancy. Available online: https://www.nhs.uk/conditions/pregnancy-and-baby/foods-to-avoid-pregnant/ (accessed on 5 September 2022).
3. NHS. Vitamins, supplements and nutrition in pregnancy. Available online: https://www.nhs.uk/pregnancy/keeping-well/vitamins-supplements-and-nutrition/ (accessed on 5 September 2022).
